# Incidental Gallbladder Cancer: Routine versus Selective Histological Examination After Cholecystectomy

**DOI:** 10.1055/s-0040-1722175

**Published:** 2021-02-01

**Authors:** Andrew Alabi, A D. Arvind, Nikhil Pawa, Shakir Karim, Jason Smith

**Affiliations:** 1 Department of Surgery, West Middlesex University Hospital, Chelsea and Westminster NHS Trust, London, United Kingdom; 2 School of Medicine, Imperial College London, United Kingdom; 3 Department of Histopathology, West Middlesex University Hospital, Chelsea and Westminster NHS Trust, London, United Kingdom

**Keywords:** cholecystectomy, histology, incidental gallbladder cancer

## Abstract

**Background** Incidental gallbladder cancer is relatively rare, with an incidence ranging between 0.19 and 5.5% of all the cholecystectomies for benign disease, and carries a poor prognosis. Currently, in the literature, there appears to be some controversy about whether all gallbladder specimens should be sent for routine histopathology. The aim of this study was to investigate the need for either routine or selective histopathological evaluation of all gallbladder specimens following cholecystectomy in our institution.

**Methods** The records of all patients who underwent a cholecystectomy (laparoscopic and open) for gallstone disease over a 5-year period (between January 2011 and January 2016) were reviewed retrospectively in a single university teaching hospital. Patients with radiological evidence of gallbladder cancer preoperatively were excluded. The notes of patients with incidental gallbladder cancer were reviewed and data were collected for clinical presentation and preoperative investigations including blood tests and radiological imaging.

**Results** A total of 1,473 specimens were sent for histopathological evaluation, with two patients being diagnosed with an incidental gallbladder cancer (papillary adenocarcinoma in situ and moderately differentiated invasive adenocarcinoma [stage IIIa]). The incidence rate was 0.14%. All patients with incidental gallbladder cancer had macroscopically abnormal specimens.

**Conclusion** Both patients in our study who were diagnosed with incidental gallbladder cancer had macroscopic abnormalities. A selective rather than routine approach to histological evaluation of gallbladder specimens especially in those with macroscopic abnormalities should be employed. This will reduce the burden on the pathology department with potential cost savings.

Symptomatic gallstone disease is one of the commonest surgical problems around the world. Approximately 10 to 15% of the western population develop gallstones, of which 1 to 4% a year become symptomatic (biliary colic, acute cholecystitis, choledocholithiasis, and gallstone pancreatitis) requiring treatment. 1 The definitive treatment of symptomatic gallstone disease is a cholecystectomy with laparoscopic cholecystectomy currently considered the gold standard treatment for patients with symptomatic gallbladder disease. 2 3 However, gallbladders could contain malignancy, which could be relatively asymptomatic at the time of diagnosis and are incidentally found on routine histological evaluation. Gallbladder cancer (GBC) is rare especially in the western population, with a recorded incidence of 0.8 per 100,000 men and 2.2 per 100,000 women in the United Kingdom. 4 It is an aggressive cancer with a poor prognosis. 5 6 Majority of cases (60–80%) of GBC is diagnosed as an incidental finding following routine histopathological examination of cholecystectomy specimen for benign disease. 7 8 Studies have shown that the incidence of incidental GBC (IGC) ranges between 0.19 and 5.5% of all cholecystectomies for benign disease. 6

Traditionally, despite the low prevalence and incidence of GBC, in the United kingdom, all gallbladder specimens regardless of its macroscopic appearance, are routinely sent for histopathological examination including our institution, which is in keeping with the recommendations of the Royal College of Pathologist report published in 2005. 9 Its primary objectives are to provide a definitive diagnosis, identify unsuspected findings, give prognostic information, guide future treatment, and document for medicolegal purposes. It also provides information for quality control and feedback for surgical decision-making.

Currently, there is a trend, which, is supported by several studies in the literature, suggesting a move away from the current practice of routine histopathological evaluation of all gallbladder specimens to a more selective approach. 10 11 This is a result of several factors including the low prevalence of IGC and economic constraints involved in the cost of processing the specimens, as well as the relative or absolute limitation in the availability of histopathological expertise.

Given this controversy, the aim of this study was to investigate the need for either routine or selective histopathological evaluation of all gallbladders following cholecystectomy in our institution.

## Material and Methods

The records of all patients who underwent a cholecystectomy (emergency and elective) for presumed benign disease between January 2011 and January 2016 were reviewed retrospectively. Cases were identified from clinical coding data as well as the histopathological electronic database. Patients who had a radiological diagnosis of GBC prior to operation were excluded from the study. In cases of confirmed GBC, the clinical notes of these patients were retrieved and data were collected regarding clinical presentation, preoperative investigations (including liver function test, abdominal ultrasound scan, computed tomography [CT] scan, and magnetic resonance cholangiopancreatography), and intraoperative findings detailed in the operation notes.

Statistical analysis of the data was performed using the Statistical Package for the Social Sciences (SPSS), version 21 (IBM Corp., Armonk, New York, United States). Quantitative variables were expressed as the median and interquartile range (IQR), and qualitative variables were expressed as frequencies and percentages.

## Results

In the study period, a total of 1,473 patients underwent a cholecystectomy, and all the specimens were sent for histopathological evaluation. The median age of these patients was 49 years (IQR: 37–63 years). The female to male ratio was 3:1. Majority of the specimens were reported as chronic cholecystitis ( *n* = 1,288 [87.4%]) ( Table 1 ). In our series, there was a patient noted to have papillomatosis with high-grade dysplasia. Two cases of IGC were found in our series, both of which were females (0.14%). The ages of these patients were 52 and 78 years, respectively.

**Table 1:** Histology of gallstone disease

Diagnosis	Number	Frequency (%)
Chronic cholecystitis	1,288	87.4
Chronic xanthogranulomatous cholecystitis	5	0.34
Gangrenous cholecystitis	9	0.61
Acute cholecystitis	17	1.20
Chronic cholecystitis with cholesterol polyps	1	0.07
Acute on chronic cholecystitis	86	5.83
Cholelithiasis	55	3.73
Papilomatosis with high-grade dysplasia	1	0.07
Adenocarcinoma	2	0.14
NAD	9	0.61
Total	1,473	

One of them was noted to have papillary adenocarcinoma in situ and the other had a stage IIIa invasive adenocarcinoma ( Table 2 ). There was no suspicion of cancer based on the preoperative radiological investigations. The patient with the adenocarcinoma in situ underwent an uncomplicated laparoscopic cholecystectomy with an R0 resection. The other patient underwent a laparoscopic converted open cholecystectomy. This patient subsequently underwent reexploration with lymph node dissection of the hepatoduodenal ligament and excision of the gallbladder bed.

**Table 2:** Clinical and pathological features of the patients with GBC

Age	Sex	Preoperative blood tests and imaging	Preoperative diagnostic	Operation	Intraoperative findings	Histology
52	F	LFTs: normal Abdominal ultrasound: thickened gallbladder wall	Acute cholecystitis	Laparoscopic cholecystectomy (emergency)	Thickened gallbladder wall	Papillary adenocarcinoma in situ
74	F	LFTs: normal Abdominal ultrasound: multiple gallstones and sludge. The gallbladder wall is slightly thick-walled. CT scan of the abdomen and pelvis: sludge and stones in the gallbladder. Intra- and extrahepatic duct dilatation.	Choledocholithiasis/acute cholecystitis	Laparoscopic Cholecystectomy and on table cholangiogram (emergency)	Thickened gallbladder wall; no stones seen within the CBD	Papillomatosis with high-grade dysplasia in a flat epithelium
78	F	LFTs: abnormal Amylase: 4,000 CT of the abdomen + pelvis: dilated CBD (11 mm) Evidence of acute pancreatitis MRCP: two stones seen within the CBD. Gallbladder was contracted and could not be assessed. ERCP: sphincterotomy, balloon trawl, and stent left in situ	Gall stone pancreatitis	Laparoscopic converted to open cholecystectomy (interval surgery)	Thickened fibrotic gallbladder wall	Moderately differentiated invasive adenocarcinoma (stage IIIa)

Abbreviations: CBD, common bile duct; CT, computed tomography; ERCP, endoscopic retrograde cholangiopancreatography; F, female; LFT, liver function test; MRCP, magnetic resonance cholangiopancreatography.

## Discussion

Although GBCs are relatively quite rare, it is the most common malignancy occurring in the biliary tract, accounting for approximately 60% of all cancers. 12 There are regional and ethnic variations in the worldwide distribution of GBC with a high incidence noted in countries such as India particularly in the northern parts, Pakistan, East Asia, Eastern Europe, and South America. 13 14 It is, however, rare in most of Northern Europe and North America. 15 Recognized risk factors for GBC include porcelain gallbladder, gallbladder polyps greater than 1.5 cm, gallstones, age > 50 years, empyema, genetic predisposition, geographical/ethnic factors, and female gender. 13 14 16

Macroscopically, GBC could present as infiltrative, papillary, nodular, or mixed form. The infiltrative form is the most common presentation and presents as thickening of the gallbladder wall. The nodular form presents as a circumscribed mass, whereas the papillary form presents as polypoid lesions with frondlike projections. 17 18

GBC carries a poor prognosis because it mostly tends to present at an advanced stage. The 5-year survival for treated cancer of less than 5%, and a reported median survival of 2 to 5 months for untreated GBC. 19 20 21 The treatment of GBC is dependent on the stage of disease at which it present. For stage I tumors (i.e., Tis and Tia), a simple cholecystectomy is considered effective treatment. The management of T1b tumors is somewhat controversial with advocates for simple and radical cholecystectomy. 21 22 23 The more advanced tumors may be treated either with radical resection provided an R0 resection could be achieved or palliation alone. 21 The benefit of such radical surgery is limited due to the associated morbidity and mortality, especially in the elderly cohort of patients. 24 25 There is no role for adjuvant therapy (chemotherapy and radiotherapy) in the treatment of advanced GBC. 21 26

The preoperative diagnosis of IGC is difficult because the clinical presentation is usually nonspecific and tends to mimic the clinical picture of benign gallstone disease. 6 This is particularly true in gallbladders containing stones, which makes the detection of a small area of thickening or mass difficult to detect on abdominal ultrasound. 27 Longstanding gallstone disease tends to cause chronic cholecystitis, which manifests as thickening of the gallbladder wall, which may also make the detection GBC even more difficult. 18

The results of our study reflect what is already known about IGC in the literature. Our incidence rate is 0.14%. Apart from a thickened gallbladder wall noted in both cases at surgery, the preoperative investigations and clinical history did not highlight any red-flag symptoms suspicious of malignancy. This is in keeping with findings from other studies, which reported that preoperative imaging and intraoperative gross findings may not be very reliable in identifying malignancy. 28 29 Lohsiriwat et al found that there were no preoperative or intraoperative findings suspicious of malignancy in 24 cases of IGC diagnosed on histological evaluation of 4,317 cholecystectomy specimens examined over an 8-year period. 16 In another study from Nepal, they found that both preoperative investigations and intraoperative examination were only accurate in identifying approximately 55% IGCs. 29 There was a trend in our study for dissection at surgery to be more difficult, leading to conversion from laparoscopic to open surgery especially in the more advanced GBC cases. No malignancy was, however, found in all the cases that had a macroscopically normal gallbladder intraoperatively and normal preoperative imaging.

There has been some concern raised about the presence of dysplasia and stage I GBC in gallbladders that appear macroscopically normal, as was seen in two cases in our series. In these cases, a simple cholecystectomy would be considered as curative and the treatment of choice. 21 23

Given the findings from our study, we suggest any patient who is over the age of 50 years, with a macroscopically abnormal gallbladder, emergency surgery, and difficult dissection leading to conversion from laparoscopic to open surgery, should have their gallbladder specimens sent for histology evaluation.

The major limitation of our study is that it is a retrospective study, which is similar to most studies on this subject matter in the literature. This introduces a heterogeneity bias, which needs to be taken into consideration when interpreting the results. Given the relatively low incidence and low numbers in most studies on IGC, a prospective multicenter study should be performed to assess the potential impact of the various criteria used for the selective approach of histological evaluation of gallbladder specimens.

In conclusion, the findings in our study reflect what has already been published in the literature in terms of IGC. Selective histological evaluation could be recommended for gallbladder specimens in patients over the age of 50 years who have macroscopically abnormal looking gallbladder, emergency surgery, and surgery with difficult dissection leading to conversion from laparoscopy to open surgery. This would potentially lead to a reduction in the cost and pathology workload and at the same time not compromise patient safety and management.
